# Identification of recruitment and retention strategies for rehabilitation professionals in Ontario, Canada: results from expert panels

**DOI:** 10.1186/1472-6963-8-249

**Published:** 2008-12-09

**Authors:** Diem Tran, Linda McGillis Hall, Aileen Davis, Michel D Landry, Dawn Burnett, Katherine Berg, Susan Jaglal

**Affiliations:** 1 Graduate Department of Rehabilitation Science, University of Toronto, Ontario, Canada; 2 Lawrence S. Bloomberg Faculty of Nursing, University of Toronto, Ontario, Canada; 3 Toronto Western Research Institute, Ontario, Canada; 4 Department of Physical Therapy, University of Toronto, Ontario, Canada; 5 Department of Health Policy and Management, University of North Carolina at Chapel Hill, USA; 6 Canadian Physiotherapy Association, Ontario, Canada

## Abstract

**Background:**

Demand for rehabilitation services is expected to increase due to factors such as an aging population, workforce pressures, rise in chronic and complex multi-system disorders, advances in technology, and changes in interprofessional health service delivery models. However, health human resource (HHR) strategies for Canadian rehabilitation professionals are lagging behind other professional groups such as physicians and nurses. The objectives of this study were: 1) to identify recruitment and retention strategies of rehabilitation professionals including occupational therapists, physical therapists and speech language pathologists from the literature; and 2) to investigate both the importance and feasibility of the identified strategies using expert panels amongst HHR and education experts.

**Methods:**

A review of the literature was conducted to identify recruitment and retention strategies for rehabilitation professionals. Two expert panels, one on *Recruitment and Retention *and the other on *Education *were convened to determine the importance and feasibility of the identified strategies. A modified-delphi process was used to gain consensus and to rate the identified strategies along these two dimensions.

**Results:**

A total of 34 strategies were identified by the *Recruitment and Retention *and *Education *expert panels as being important and feasible for the development of a HHR plan for recruitment and retention of rehabilitation professionals. Seven were categorized under the *Quality of Worklife and Work Environment *theme, another seven in *Financial Incentives and Marketing*, two in *Workload and Skill Mix*, thirteen in *Professional Development *and five in *Education and Training*.

**Conclusion:**

Based on the results from the expert panels, the three major areas of focus for HHR planning in the rehabilitation sector should include strategies addressing *Quality of Worklife and Work Environment*, *Financial Incentives and Marketing *and *Professional Development*.

## Background

Demand for rehabilitation services is expected to increase within the next decade primarily due to factors such as an aging population, workforce pressures, rise in chronic and complex multi-system disorders, advances in technology, and changes in health service delivery models [1-4]. In Canada, rehabilitation personnel constitute the third largest health professional group after nurses and physicians. Despite the size of this workforce, studies have consistently reported ongoing shortages of physiotherapists (PTs), occupational therapists (OTs) and speech-language pathologists (SLPs) across all jurisdictions [5-7]. Similarly, recruitment and retention of rehabilitation professionals has been considered a challenge internationally, nationally and provincially. At the international level, the literature reports recruitment and retention difficulties of rehabilitation therapists in countries such as Australia, New Zealand, United Kingdom and the United States [8-13]. Provinces across Canada face similar issues; with Ontario projected to face the most difficulty due to its population growth rate [14].

Based on the Canadian Institute for Health Information's *Health Personnel Trends in Canada from 1993 to 2002 *report, numerous factors have been suggested to influence demand for physiotherapy and occupational therapy services. Factors that may influence increase demand for physiotherapy include: shift in health service delivery models from hospital to community care; earlier patient discharge; increased expectations from aging Canadians concerning more active lifestyles; growing private practice sector and continued shortages for PTs in both private and public sectors in rural, remote and urban settings across Canada [2]. In 1993, an Ontario study stated that in order to meet demands of changing health care policy, medical technology and demographic changes in the population, the PT profession required an annual growth rate of 4.4% until the year 2000 [5]. However, the national health personnel databases revealed that the actual average annual growth rate of active PTs in Canada from 1995 to 2004 was only 2.5%, approximately half of the projected requirement suggested to meet demand [15].

Similarly, in Ontario in the early 1990s an increase in demand for OTs was projected because of the reported shortage in OTs and high attrition rate [16]. The shortage of OTs was explained by another Ontario study to be the result of the changing philosophies of care and management for the disabled, and a clearer understanding of the role of OT in the physical and mental well-being of the disabled [17]. In terms of actual shortages, some authors have reported ongoing vacancies and recruitment difficulties for OTs [11,18] while others have reported an increase in demand for both PTs [19] and OTs [20].

Speech language pathology is facing similar service demands. A report released in March 2003 by the College of Audiologists and Speech-Language Pathologists of Ontario (CASLPO) concluded that based on prevalence rates for Ontario residents with speech, language and related disorders, the demand for service would increase by 13% while the number of SLPs would decrease by 4% resulting in an overall reduction in service of 15% [21]. The American Speech-Language-Hearing Association (ASHA) has been tracking SLP vacancies. In their 2005 ASHA Speech-Language Pathology Health Care survey, 48% of respondents indicated that they had funded unfilled positions for SLPS in their agency [22]. The same survey also reported that 65% of respondents in home care indicated that job openings were more numerous than job seekers in their geographic area.

While labour market demand and supply are influential factors on recruitment and retention decisions, the development of strategies requires an understanding of conceptual frameworks or theories to categorise and explain how other underlying factors impact health worker's mobility. For example, Lehmann et al.'s model described that health worker's decisions to accept and stay in remote areas in the public sector depends on two interrelated aspects: the impact of the different environments (i.e. individual, local, work, national and international) and the location of decision-makers (i.e. local government, Ministry of Health, HR directorate, public service and other ministries)[23]. Behavioural and social science theories, such as those explained by Tett and Meyer, found that job satisfaction and organizational commitment each contribute independently to the prediction of the intention to resign (turnover), however job satisfaction was a stronger predictor than organizational commitment[24]. Based on this notion, considerable research has been devoted to identifying factors that affect job satisfaction among rehabilitation professionals. While there is no single, agreed upon model of job satisfaction, a variety of theoretical models have been studied to explain concepts and relationships associated with overall job satisfaction. The two most commonly used theories of job satisfaction for rehabilitation professionals are the Herzberg's Motivation-Hygiene Theory[25] and Mottaz's concepts of work values and work rewards [26].

A number of rehabilitation studies have used the Herzberg's Motivation-Hygiene Theory, also known as the two-factor theory of motivation to explain associations between motivation, job satisfaction and retention factors among OTs, PTs and SLPs [27-31]. Frederick Herzberg et al. explained that there were two independent incidents occurring at peoples' jobs: one that made them feel good or satisfied, and another that made them feel bad or dissatisfied at work [25]. Intrinsic factors that motivate people such as achievement, recognition, work itself, responsibility, advancement and personal growth were called the "motivators" which lead to feelings of satisfaction. Extrinsic factors such as work conditions, company policies, supervision, interpersonal work relations, salary and job security, known as "hygiene" factors, were claimed to prevent dissatisfaction. "Motivators" directly affect a person's motivational drive to do a good job, therefore they are believed to be more important than hygiene factors.

Mottaz on the other hand, accounted for individual differences in job satisfaction among workers and based his study on two dimensions: "work rewards" and "work values"[26]. "Work rewards" are perceived characteristics of the job and have three conceptual clusters which include task, social and organizational rewards [26]. Mottaz describes "task rewards" (intrinsic) as having five independent characteristics including: skill variety, task identity, task significance, autonomy and feedback. Examples include interesting and challenging work, self-direction and responsibility, creativity, opportunities to use one's skills and feedback. In the same study, Mottaz stated that "social rewards" (extrinsic) are derived from the interpersonal relationships established with others at work. Having supportive colleagues and supervisors is an example of this dimension. Lastly, "organizational rewards" (extrinsic) are tangible rewards that are provided by the employer/organization to facilitate performance. Such factors include working conditions, pay and fringe benefits, career advancement and security. The second dimension of job satisfaction is based on "work values", which is the importance that individuals place on their work rewards [26]. For example, some rehabilitation therapists may value extrinsic rewards such as pay and benefits as more important than intrinsic factors like clinical autonomy and challenging work. Although the Herzberg and Mottaz conceptual frameworks are organized differently, their job satisfaction variables are very similar (i.e. work conditions, pay, interpersonal relationships, etc.) and they both classify these factors as having intrinsic or extrinsic elements.

Despite the growing body of literature on recruitment and retention factors in various industries, there is a minimal amount of research studying these factors specifically among rehabilitation professionals. One published study however, did look at extrinsic and intrinsic job satisfaction factors on recruitment and retention of rehabilitation professionals (OTs, PTs and SLPs)[32]. Results from this study showed that intrinsic factors such as professional growth and having a work environment in line with personal values are more significant in predicting career satisfaction than extrinsic factors such as pay and continuing education. These same intrinsic factors are also significant in predicting retention in rehabilitation professionals. Another study looking at recruitment and retention of allied health professionals in the rural areas in New South Wales identified that the main reasons why people liked working in rural areas were because of the attractive environment and helpful team members[33]. However 82% of employees reported that having their partner move away was the number one reason for leaving a rural job. A similar study was conducted among OTs and PTs in Northwestern Ontario[34]. Findings from this study indicated that factors contributing to initial decision on location of practice include availability of leisure/recreation activities, proximity of family origin and influences of spouse/partners. Study results also showed that the main reasons therapists left their job were to be closer to their family, lack of job opportunity and spousal influence.

Solely understanding factors that influence recruitment and retention decisions is not sufficient in the development of a HHR plan for rehabilitation professionals. In order for the plan to be effective and sustainable in addressing these factors, the most important and feasible workforce strategies needs to be identified.

There have been a number of reports on health human resources (HHR) planning, recruitment and retention strategies for physicians [35,36] and nurses [37], however information regarding rehabilitation professionals is lacking. Canadian reports indicate that the main reason for significant gaps in this field is the absence of current and reliable data available on supply, demand and labour force participation trends for rehabilitation therapists [38-40]. There is some research that has investigated theoretical models of job satisfaction on recruitment and retention [28,32]; however few studies have looked at how these models have been implemented. Other studies have examined the relationship of gender, workplace setting (i.e. hospital, ambulatory, rehabilitation, acute and long-term care) and geographical location (i.e. rural or urban) on job satisfaction and retention among rehabilitation professionals [41-43]. Furthermore, no empirical studies have examined conceptual frameworks for organizing recruitment and retention strategies for rehabilitation therapists. To address this gap, this research identified recruitment and retention strategies from the literature for rehabilitation professionals and determined their importance and feasibility using expert panels.

## Methods

### Phase 1: Literature Review

#### Identification of recruitment and retention strategies

A review of the literature was conducted to identify recruitment and retention strategies for rehabilitation therapists. In this study, rehabilitation professionals were defined as physical therapists (PTs), occupational therapists (OTs) and speech-language pathologists (SLPs). Both published and non-empirical literature was accessed in this review. Keywords used to search for relevant published studies in the Consolidated International Nursing and Allied Health Sciences Library (CINAHL) (1982 to 2005) and Medline (1996 to 2005) included: "health human resources or health manpower", "rehabilitation or rehabilitation professionals or vocational", "allied health professionals or personnel", "recruitment strategies", "retention strategies", "physical therapist or physiotherapist", "occupational therapy or occupational therapist", "speech-language pathologist or speech-language pathology". Non-empirical literature searches were made on international and national on-line catalogues and publications from health organizations, professional associations, and hospital and home care organizations. International reports were limited to developed countries since the purpose of this study was to identify strategies appropriate to the Ontario setting.

#### Organization and consolidation of strategies

There was a paucity of peer-reviewed studies obtained exploring rehabilitation HHR strategies, therefore the majority of strategies were selected from grey literature reports from international, national and provincial health organizations. From the literature review, 107 potential strategies were identified according to their relevance to HHR issues for rehabilitation in Ontario. These strategies were then categorized into two broad groups: A) *Recruitment and Retention *(n = 73), and B) *Education *(n = 34). The majority of strategies were not specific to rehabilitation professionals and they were reviewed by a group of three individuals collectively (rehabilitation researcher, manager, and clinician) for duplication, clarity, action focused properties and appropriateness to the Canadian or Ontario setting. When necessary, a small number of strategies were re-worded to be relevant to a rehabilitation context. This analysis resulted in the selection of 40 *Recruitment and Retention *and 24 *Education *strategies. Only 14 recruitment and retention and six education strategies were obtained from peer-reviewed articles. Since the majority of strategies were identified from the grey literature, it was not surprising that there was no apriori peer-reviewed conceptual framework that reflected the breadth of the strategies obtained from the literature review. As a result, the themes used by the Health and Community Services Human Resources Sector Study in Newfoundland and Labrador [44] formed the basis for the organizational framework for this study since they aligned with the identified strategies. Each group was further categorized into the five themes (three for *Recruitment and Retention *and two for *Education*). The three *Recruitment and Retention *strategy themes were: (1) *Quality of Worklife and Work Environment *[n = 19]; (2) *Workload and Skill Mix *[n = 6]; and (3) *Financial Incentives and Marketing *[n = 15]. The two *Education *strategy themes were: (1) *Education and Training *[n = 11] and (2) *Professional Development *(n = 13).

### Phase 2: Expert Panel

#### Participant Selection

Once this study was approved by the Research Ethics Board at the University of Toronto, key informants who participated in a previous study regarding rehabilitation supply and demand at the University of Toronto [21] were asked to nominate potential participants for the panels. The selection criteria considered were acknowledged leadership in the panel member's specialty, expertise in recruitment and retention or education and training of rehabilitation professionals. Absence of conflicts of interest, geographic diversity, and diversity of practice setting were also considered. After purposefully selecting the initial list of candidates from among the nominations, each nominee was contacted to establish their interest and availability. Those who expressed an interest in participating were asked to send their curriculum vitae to help the research team evaluate their contributions to their field of expertise. Once candidate panelists were selected, each received a letter explaining the expert panel process and consent form. Two separate panels were constructed: one for *Recruitment and Retention *(n = 8) and the other for *Education *(n = 9) (Table 1). The size of the panel was large enough to permit diversity of representation while still being small enough to allow all participants to be involved in the group discussion [45].

**Table 1:** Expert panel constituency

**Panel A:** **Recruitment and retention panel**	**Number of participants**
HHR strategic advisory member	2
Professional association HR policy expert	2
HHR consultant	1
Rehabilitation hospital recruiter	1
HHR researcher	1
Home care service provider employer	1

**Panel B:** **Education panel**	**Number of participants**

Clinical educators from various rehabilitation professions	4
Health service researchers	2
Human resource policy experts from professional rehabilitation associations	2
Education administrator	1

#### Expert Panel Process: Round 1 Survey

A modified-delphi technique was then used for the expert panel process, [46,47] based on the RAND/UCLA appropriateness method [45]. In round 1, members of the *Recruitment and Retention *panel were sent an electronic survey containing the 40 strategies identified from the literature review and the *Education *panelists were also sent an electronic survey with 24 strategies. For Round 1, each panel was asked to rate the strategies using a nine-point Likert-type rating scale that ranged from "none" (1) to "maximum" (9), on two key dimensions: *Feasibility *and *Importance*. *Feasibility *was defined as the practicality and cost implications of the strategy and was rated from the respondents' perspective. *Importance *was defined as how valuable, appropriate and useful the strategy could be for rehabilitation HHR planning in Ontario. At the end of the survey, panelists were given the opportunity to suggest additional strategies that they felt were appropriate to consider. Once completed, panelists were asked to return the survey to the study office by email or fax prior to the expert panel meeting in Round 2. Data from each questionnaire were entered into a spreadsheet and tabulated. Descriptive statistics were calculated for each strategy using frequency distributions and proportional percentages of respondents. Importance and feasibility rankings were based on the percentage of expert panelists' low, medium and high ratings.

#### Expert Panel Process Round 2: Expert Panel one-day meeting

After the independent completion of the survey, each panel was convened separately for a one-day meeting for final discussions, debates and consensus voting to decide on strategies [48]. A strategy that had been scored 7, 8 or 9 for both feasibility and importance by two-thirds of the panel was considered a high rating. Strategies that had a combination of medium and high scores between 4 and 9 in either of the two dimensions were considered medium rated strategies, while low rated strategies had scores between 1 and 3 for both dimensions.

On the day of the meeting, the panelists were given a copy of the aggregated survey results indicating the ratings of all of the strategies. High and low rated strategies were not discussed as there was already consensus, whereas all medium rated strategies were subject to discussion. Using a nominal group process [47], each strategy was discussed in turn, and panelists were given an opportunity to raise any issues or concerns regarding the clarity and wording of each strategy. Each of the strategies discussed were then individually rated a second time by each panelist in an attempt to reach further consensus.

## Results

### Selection of strategies for Round 1: Modified Delphi process

Following Round 1 rating of the 40 identified strategies, the *Recruitment and Retention *panel reached consensus on 12 strategies. However, 14 had a combination of high/medium importance and feasibility ratings and 14 had medium ratings on both dimensions, therefore it required further discussion. An additional strategy regarding family relocation programs was added by this panel.

The *Education Panel *ranked 16 of 24 strategies with high importance and feasibility after Round 1. Since there were only eight strategies with medium ratings, this expert panel decided to review all the strategies at the face-to-face meeting to discuss the rationale that would explain why some of the highly rated strategies were not already implemented and to come to a consensus on the other eight medium rated strategies. They also added an additional strategy for career paths.

### Selection of strategies for Round 2: Face-to-face meeting

A total of 34 strategies were identified by both the *Recruitment and Retention *and *Education *expert panels as being important and feasible for the development of a HHR plan for recruitment and retention of rehabilitation professionals. Under the *Recruitment and Retention *theme, seven were categorized as *Quality of Worklife and Work Environment; *two were *Workload and Skill Mix*, and another seven were *Financial Incentives and Marketing*. As for the *Education *panel, five were categorized as *Education and Training *strategies while the other thirteen were related to *Professional Development*.

As indicated in Table 2, at the end of the second round of voting, the *Recruitment and Retention *panel had a total of 16 highly important and feasible strategies, 8 high/medium importance and feasibility, 8 medium and 9 low ratings for both dimensions. The *Education *panel on the other hand had a total of 18 high, 1 high/medium, 3 medium and 3 low rating strategies.

**Table 2:** Strategies by theme

	Importance and Feasibility Ratings				
	
	**N**	High (%)	High/Med (%)	Med (%)	Low (%)
**Recruitment & Retention**					

Quality of Worklife & Work Environment	19	7 (44)	7 (88)	1 (12)	4 (44)
Workload & Skill Mix	6	2 (12)	1 (12)	3 (38)	0
Financial Incentives & Marketing	16	7 (44)	0	4 (50)	5 (56)

**TOTAL**	**41**	**16**	**8**	**8**	**9**

**Education**					

Education & Training	11	5 (28)	1 (100)	3 (100)	2 (67)
Professional Development	14	13 (72)	0	0	1 (33)

**TOTAL**	**25**	**18**	**1**	**3**	**3**

### Recruitment and Retention Strategy Rankings

Table 3 provides a detailed description and ranking of each of the recruitment and retention strategies that were rated highly important and feasible. The importance and feasibility rankings were based on the largest proportion of panel members rating a strategy a 7, 8 or 9. The overall combined ranking was based on the average of the proportion of these two dimensions. Among these selected strategies, the majority were classified under *Quality of Worklife and Work Environment *(44%) and *Financial Incentives and Marketing *(44%), followed by *Workload and Skill Mix *(12%). It should be noted that some strategies had equal rankings; therefore the total number of rankings did not equal the total number of strategies.

**Table 3:** Recruitment and retention strategies with high importance and feasibility in order of overall ranking

**RANKING**				
**O**	**I**	**F**	**Theme**	**RECRUITMENT AND RETENTION STRATEGIES**

1	1	1	Quality of Worklife & Work Environment	**Personal safety **Improve and maintain the safety of rehabilitation professionals by reducing aggression abuse and violence in the workplace. Examples could include: zero tolerance polices; and access to employee support programs and providing assistance to rehabilitation professionals who work alone, particularly in rural environments[37].

2	2	2	Financial Incentives & Marketing	**Professional development in budget planning **Make professional development (PD) a regular part of budget planning [63].

3	2	3	Quality of Worklife & Work Environment	**Communication between employer and worker **Ensure open and timely communication between employer and worker. For example communication between staff supervisors and upper management is encouraged via open door policies, employee advisory committees and regular staff meetings and evaluations [51].

4	1	5	Quality of Worklife & Work Environment	**Training/growth opportunities **Provide training and growth opportunities. Examples could include: continuing education and re-certification requirements paid for by the organization; educational leaves; mentorship programs; internal job fairs; job-share arrangements; and secondments to other areas or agencies [51].

5	2	4	Quality of Worklife & Work Environment	**Tangible resources **Ensure that workers have the tangible resources to do their job. For example provide computers and specialized equipment telephone systems vehicles office space meeting rooms and well-maintained workplace facilities [51].

5	2	4	Financial Incentives & Marketing	**Rural and remote orientation packages **For rural and remote communities ensure comprehensive orientation packages are made available to potential recruits [64].

6	6	2	Financial Incentives & Marketing	**Increase public awareness of rehabilitation careers **Position the rehabilitation profession as an attractive career choice by developing comprehensive province-wide web and print education/awareness campaign to heighten the younger generations' and the public's awareness [53].

6	4	3	Financial Incentives & Marketing	**Increase high school student awareness of rehabilitation careers **Position the rehabilitation profession as an attractive career choice by developing high school co-op programs volunteer service programs and job shadowing programs to cultivate prospective recruitment relationships and to capitalize on high school student community service programs [53].

7	2	7	Quality of Worklife & Work Environment	**Work safety **Improve and maintain the safety of rehabilitation professionals by minimizing the susceptibility to work-related injury. Examples could include: designing ergonomically sound work environments; providing patient lifting equipment; and ensuring adequate staff support during patient mobilization, lifts and transfers [37].

8	3	4	Quality of Worklife & Work Environment	**Workplace audit **Develop a workplace audit exercise/assessment tool that evaluates current workplace processes and determines which practices/issues enhance and deter employee recruitment satisfaction and retention of rehabilitation professionals [53].

9	6	3	Financial Incentives & Marketing	**Employer/workplace awards **Create and present a special award for organizations that excel in originality and innovation as employers/workplaces of choice [53].

9	4	4	Financial Incentives & Marketing	**Family relocation programs **Implement family relocation programs to target difficult to supply areas in order to recruit rehabilitation professionals (added by panel).

10	4	5	Financial Incentives & Marketing	**Competitive wage packages **Provide competitive compensation packages for wages. Examples could include: fair wages using wage grids which recognize education and experience levels; renegotia-ting work terms following skills upgrading; and refining internal wage equity (i.e. aligning the compensation of equivalent positions across areas) [51].

11	5	6	Quality of Worklife & Work Environment	**Congruence between employer and staff values **Demonstrate congruence between agency/employer and staff values. Examples could include: being clear about the organization's philosophy expectations and work culture; and involving fellow team members the service recipient and family members in the hiring process if appropriate [51].

11	6	5	Workload & Skill Mix	**Caseload management database **Implement a computerized database system with accessible real-time information to facilitate caseload management [57].

12	7	5	Workload & Skill Mix	**Support personnel **Use support personnel to increase efficiency of utilization of scarcer and higher order rehabilitation competencies (i.e. physiotherapy assistants or exercise therapists) [64].

O = Overall combined importance and feasibility ranking

I = Importance ranking

F = Feasibility ranking

Recruitment and retention strategies that had a combination of high and medium ratings in either of the two dimensions included: sense of empowerment in promoting healthy work environments; team-building exercises; developing participatory decision-making systems; improving rural working conditions; recognizing work-life balance; creating an environment where staff are valued; optimizing scope of practice and work-management autonomy. Strategies that had medium importance and feasibility ratings included: resolving concerns about liability and accountability in collaborative practice; recruiting international trained therapists; opportunity to work in different settings; interprofessional payment schemes; family leave; staff recognition and creating a position for a provincial health professional recruiter. Low importance and feasibility strategies included: word of mouth references; bursaries and retention bonuses; exchange employment opportunities; health promotion; retention workshop; 80–20 staffing model (80% clinical and 20% learning new skills or training others); using recruitment agencies and providing recruitment bonuses (Table 4).

**Table 4:** Recruitment and Retention Strategies with Medium or Low Importance and Feasibility

**O**	**I**	**F**	**Recruitment and Retention Area**	**STRATEGIES**	**Theme**
13	12	8	Sense of empowerment	Focus on sense of empowerment by involving rehab professionals in the development of strategies that promote healthy work environments.	Quality of Worklife & Work Environment

14	8	12	Team work	Develop positive relationships between staff by way of team-work and team-building exercises.	Quality of Worklife & Work Environment

14	8	12	Participatory decision-making systems	Develop participatory decision-making systems. This could include: adopting team-models of work; flattening organizational hierarchies; and decentralizing authority.	Quality of Worklife & Work Environment

15	13	9	Rural working conditions	Improve working conditions for rural rehab professionals. Examples could include: providing a coordinated solution to assist with coverage for leave and continuing education; and reducing unreasonable caseload and travel expectations.	Quality of Worklife & Work Environment

16	9	10	Work-life balance	Recognize balance between the employee's work and family demands. Examples could include: liberal and flexible vacation and time-off policies; and employee requests for change in work schedules to enable personal commitments to be met.	Quality of Worklife & Work Environment

16	9	11	Feeling valued as an employee	Create an environment where all staff are valued for their knowledge skills and personal qualities. Examples could include: developing effective staff involvement activities; and providing opportunities for all staff to develop personally improve their skills gain relevant qualifications to progress.	Quality of Worklife & Work Environment

17	10	10	Optimize scope of practice	Enhance opportunities for professionals to work to optimal scope of practice. This will ensure the system's capacity to meet local patient and population health needs.	Workload & Skill Mix

18	11	13	Work-management autonomy	Provide flexible work-management autonomy. Examples could include: giving employees the independence (and accountability) to implement what they feel are the best solutions to the challenges they face; enabling staff to work with a variety of clients in diverse settings; and changing caseload if appropriate.	Quality of Worklife & Work Environment

19	14	14	Collaborative practice liability and accountability	Professional liability protection organizations, government regulators and patient safety organizations should resolve concerns about liability and accountability in collaborative practices. This will enhance patient safety risk management and teamwork in collaborative practice environments.	Workload & Skill Mix

20	16	14	Recruit international trained therapists	Recruit overseas trained rehab professionals.	Workload & Skill Mix

21	15	15	Junior rotational scheme	Develop a junior rotational scheme giving staff the opportunity to work in different settings (i.e. acute and community).	Workload & Skill Mix

22	16	16	Competitive benefit packages	Provide competitive compensation packages for benefits. Examples could include: providing comprehensive benefits packages; increasing contribution by the employer to benefit packages for longer-term employees; and offering the same benefits to all staff regardless of employment terms.	Financial Incentives & Marketing

23	17	18	Interprofessional payment schemes	Accelerate the shift to provider payment schemes that stimulate interprofessional teamwork.	Financial Incentives & Marketing

24	18	19	Family leave	Improve and maintain the health of rehab professionals by employing strategies to address absenteeism. Examples could include: providing assistance with childcare and eldercare; offering life and career counseling sabbaticals or temporary leaves; and access to recreation facilities and other mechanisms for stress reduction.	Quality of Worklife & Work Environment

25	20	17	Staff recognition	Develop staff recognition programs/initiatives. Examples could include: developing long-term service awards and/or achievement awards; and providing social outings annual BBQs and holiday parties.	Financial Incentives & Marketing

25	19	20	Provincial health professional recruiter	Develop a Provincial Health Professional Recruitment position (i.e. act as a provincial representative at job fairs implement a provincial web-site for recruitment develop marketing tools etc.)	Financial Incentives & Marketing

26	21	21	Word of mouth	Network with other agencies professional groups business associations and chamber of commerce to advertise jobs find recruits via word-of-mouth references and identify human resources that can be shared.	Financial Incentives & Marketing

26	21	21	Bursary programs and Retention bonuses	Create bursary programs and retention bonuses to target difficult to recruit rehab professionals in locations throughout the province.	Financial Incentives & Marketing

27	21	24	Exchange employment opportunities	Investigate rotating exchange employment opportunities in workplaces where there is little opportunity for change (permit health professionals opportunity to work in an alternative setting for a selected time period).	Quality of Worklife & Work Environment

28	22	22	Health promotion	Improve and maintain the health of rehab professionals by developing innovative health promotion strategies. For example provide facilities and counseling to rehab professionals to assist with their healthy lifestyles.	Quality of Worklife & Work Environment

29	23	22	Retention workshops/conferences	Present an interactive conference/workshop on the concept of retention management featuring innovative retention practices. This conference should identify best practices and research-based strategies on organizational programs and policies that can increase hospital retention rates.	Quality of Worklife & Work Environment

30	23	23	80–20 staffing model	Evaluate the 80–20 staffing model for rehab professionals over age 55 in hospitals (i.e. Spend 20% of their time on learning new skills and training others and 80% doing clinical work).	Financial Incentives & Marketing

31	23	24	Research on work environments and lifestyle	Improve and maintain the health of rehab professionals by conducting research regarding their work environments and lifestyles and their effect on the physical and mental health status.	Quality of Worklife & Work Environment

32	24	24	Recruitment agencies	Use professional recruitment agencies especially during periods of high turnover.	Financial Incentives & Marketing

33	24	25	Recruitment bonuses	Provide recruitment bonuses for employees who recommend people who subsequently get hired by the agency.	Financial Incentives & Marketing

O = Overall Ranking; I = Importance Ranking; F = Feasibility Ranking

### Education Strategy Rankings

Education strategies that were rated highly important and feasible are described in Table 5. The majority of strategies in this group tend to be in the area of *Professional Development *(72%), more so than *Education and Training *(28%).

**Table 5:** Education Strategies with High Importance and Feasibility in Order of Overall Ranking

**RANKING**				
**O**	**I**	**F**	**Theme**	**EDUCATION STRATEGIES**

1	1	1	Professional Development	**Preceptorship training **Provide preceptorship training (e.g. problem-based learning) to boost the quantity and quality of student placement to improve the quality of preceptor worklife[65].

1	1	1	Professional Development	**Rural and remote mentors **Ensure access to professional colleagues/mentors for newly graduated practitioners in rural and remote locations through use of innovative communications technology [44].

1	1	1	Professional Development	**Career paths **Develop career paths within individual professions/promotion of opportunities within health care facilities for such career paths (added by panel).

2	1	2	Professional Development	**Minimize rural and remote isolation **Minimize the professional and personal isolation of rural and remote rehabilitation personnel by establishing formal and informal networks. These networks could be used to provide clinical and professional mentoring supervision and support [64].

2	1	2	Professional Development	**Access to research information **Ensure access to professional academic and research information through various media. Examples could include: computer and internet access and subscriptions to relevant professional journals and literature [44].

3	1	2	Professional Development	**Flexible delivery in continuous professional development **Incorporate professional group needs; adult learning principles and flexible delivery in the provision of continuous professional development [65].

4	2	2	Education & Training	**Rural and remote continuing education **Develop and implement innovative programs that deliver continuing education to rehabilitative health care personnel located in remote locations [66].

4	2	2	Education & Training	**Financial support for rural and remote clinical placements **Build on existing mechanisms to expand the availability of rural and remote clinical placements by providing financial and accommodation support/incentives [64].

4	1	3	Professional Development	**Continuing education **The Ministry of Health of Ontario (MOHLTC) should expand capacity for continuing education in all Ontario Hospital types and settings [53].

5	3	4	Education & Training	**Rural practice education stream **Realign curriculum to close the gaps between classroom education and the realities of rural practice. For example incorporate a dedicated rural practice focus/stream using innovative interdisciplinary approaches [66].

6	4	3	Education & Training	**Clinical placements **The Ministry of Health of Ontario (MOHLTC) should expand capacity for student clinical placements and tuition reimbursement programs in all Ontario hospital types and settings. Examples could include: earmarked funds; strategies in hospital corporate plans and working in collaboration with other stakeholder groups [53].

6	2	3	Education & Training	**National standardized assessment for international trained therapists **Develop and implement nationally standardized assessment processes to enable the integration of international graduates wishing Canadian licensure in regulated rehabilitation professionals [63].

6	3	3	Professional Development	**Rural practice scholarships **Consider additional means of recognizing rural practice such as formal qualifications/certification and additional scholarships ensuring greater access to professional development opportunities by existing rural practitioners [64].

7	2	5	Professional Development	**Percentage payroll to professional development **Dedicate a minimum percentage of payroll (i.e. 0.4%) to professional development priorities not included in the Conference Board of Canada criteria such as attending external conferences and workshops specialty and advanced education and improving access to research information and colleagues provincially nationally and internationally [44].

7	2	5	Professional Development	**Competency-based education training **Collaborate with other employers independent providers and other Sectors to actively support competency-based education training and professional development [67].

7	2	5	Professional Development	**Rural and remote teaching and training activities **Facilitate the involvement of rural and remote rehabilitation professionals in student research projects formal teaching and training and informal learning activities [64].

7	5	5	Professional Development	**Clearinghouse resource centre **Establish a clearinghouse and resource centre that is easily accessible 24 hours a day/7 days a week to assist rural and remote rehabilitation professionals with clinical and management issues [64].

8	6	6	Professional Development	**Community-based professional development **Promote community-based professional development opportunities through greater use of distance education and teleconferencing. Examples could include: journal clubs; telehealth discussion groups; and writing articles for publication in newsletters and journals [44].

O = Overall combined importance and feasibility ranking

I = Importance ranking

F = Feasibility ranking

The medium rated education strategies included: expand interprofessional education; provide incentives for students interested in rural practice; summer mentorship programs for high school students; and aboriginal student support program. The strategies that were considered neither feasible nor important included: using return of service contracts after professional development; create a tiered pathway approach through modular education and laddered credentialing and in accreditation standards allow greater use of rural practice sites (Table 6).

**Table 6:** Education Strategies with Medium or Low Importance and Feasibility

**O**	**I**	**F**	**Education Area**	**STRATEGIES**	**Theme**
9	7	8	Interprofessional education	Expand opportunities for interprofessional education. Examples could include: using funding to stimulate change; collaborating between ministries of health and education; ensuring that academic and clinical training sites are both supportive and supported; and making sure there are collaborative practice environments where students can train and work.	Education & Training

10	8	10	Preferred admission for rural practice	Provide specific incentives including admissions preferences for students interested in rural practice.	Education & Training

11	10	9	High school student summer mentorship programs	Entry to-practice rehabilitation programs should offer summer mentorship opportunities for promising local high school students to experience the university and to explore the realm of professional education while earning credit towards high school graduation.	Education & Training

12	9	10	Aboriginal support program	Create a multi-professional support program for Aboriginal students. For example a college or university could work with a remote community to educate a group of students in a range of health professions which the community needs.	Education & Training

13	11	11	Return of service contracts	Ensure a return on investment in continuing professional development and skills upgrading through the use of Return of Service Contracts.	Professional Development

14	12	12	Rural and remote tiered pathway	In rural and remote areas create a tiered pathway approach through modular education and laddered credentialing to provide students with the option to graduate into the health care workforce at various stages of training.	Education & Training

15	13	13	Flexible accreditation of rural practice	Provide sufficient flexibility in accreditation standards to allow greater use of rural practice sites as part of the professional education process.	Education & Training

O = Overall Ranking; I = Importance Ranking; F = Feasibility Ranking

Since the purpose of the panel was to identify recruitment and retention and education strategies that could inform the development of a HHR plan for rehabilitation professionals, there was also discussion about contextual factors that would influence a plan. Panelists commented that key factors to consider prior to implementation of these strategies should include workplace setting, geographical location (i.e. urban and rural) and gender issues.

## Discussion

The purpose of this study was to identify recruitment and retention strategies to inform the development of HHR planning for rehabilitation professionals in Ontario. This study highlights that *Quality of Worklife and Work Environment*, *Financial Incentives and Marketing *and *Professional Development *are the three major areas of focus when developing a competitive HR plan in the rehabilitation sector.

### Quality of Worklife and Work Environment

*Quality of Worklife and Work Environment *strategies ranked among the top category for recruitment and retention of rehabilitation therapists. This has also been found among nurses [44] where it was reported that addressing such factors can affect the overall success of the program [49]. Specifically, our findings showed that the top ranked strategy for both importance and feasibility was improving and maintaining the safety of rehabilitation professionals in the workplace. Specific strategies that could reduce aggression, abuse and violence in the workplace include: zero tolerance policies, access to employee support programs and providing assistance to rehabilitation professionals who work alone (i.e. home care and rural and remote areas). Since there is less control over the environment in the home care setting, safety may become a greater concern in one practice setting over another. This might suggest that because there is less control in environments such as home care, remote areas, and psychiatric settings, maintaining safety will be more difficult and that solutions will need to be tailored to these settings in order to ensure retention of providers. Although no studies have looked at implementing personal safety strategies for home care therapists, written policies and procedures for home care nurses during inclement weather and for dealing with abusive or dangerous patients, families and neighbourhoods have been reported [50].

In addition to the above, ensuring open and timely communication between employer and worker was also ranked highly among the recruitment and retention strategies. Examples of strategies include: open door policies, employee advisory committees and regular staff meetings and evaluations. Although these strategies were reported to be used among organizations providing services to persons with developmental disabilities in Alberta, there was no description of the organizations, sample size or methodology [51]. Similar findings were found in a qualitative study among 16 nurses working from diverse practice settings (acute, long-term care, rehabilitation and community; from both urban and rural areas) in a health region in western Canada. From the semi-structured interviews, study participants expressed a desire for improved consultation and communication with nurses regarding changes to the health care system [52].

### Financial Incentives and Marketing

Another area that was ranked highly was marketing strategies to increase high school student and public awareness of rehabilitation careers. These specific strategies were also recommended by the Ontario Hospital Association (OHA) in order to establish a competitive position for Ontario hospitals in respect of recruitment and retention of health care professionals [53]. Similar strategies have been developed by the American Physical Therapy Association (APTA) in response to the declining number of students applying to Physical Therapy Education Programs [54]. To address this trend, APTA developed a campaign to promote Physical Therapy as the profession of choice to high school and college students across the United States. The potential components of their plan included: developing a "Recruiting Kit" for educators, students and various APTA members to be used in the high school and college settings to introduce Physical Therapy as a career; establishing public relations initiatives that demonstrate the role of Physical Therapy in the public arena targeting minority groups that are underrepresented in the profession; and creating alliances with professional associations of high school guidance counsellors and educators. Based on our finding, the APTA model may have applicability in Ontario.

### Workload and Skill Mix

Of the six *Workload and Skill Mix *strategies only two were highly rated: implementing a caseload management database and using support personnel. Caseload management has been identified in the literature as an issue affecting all three rehabilitation professions. For example, in physiotherapy, Christie's study [55] found that caseload expectations tended to be significantly higher than the reality and that caseload varies across different programs. Similarly, the Canadian Association of Speech-Language Pathologists and Audiologists (CASPLA) survey indicated that factors affecting the workload of SLPs include delivery models, client disorder, severity and work setting [56]. A literature review and environmental scan undertaken by the Canadian Association of Occupational Therapists (CAOT) proposed that guiding principles for caseload management should include: evidence-based occupational therapy, cost-effectiveness, accountability, professional leadership and expert judgment, comprehensiveness and flexibility [57]. Therefore, upon implementation of a caseload management database for rehabilitation, key factors to consider include workplace setting and client service delivery models.

The other highly rated *Workload and Skill Mix *strategy was the use of support personnel (i.e. physiotherapy assistants or exercise therapists) to increase efficiency of utilization of scarcer and higher order rehabilitation competencies. Considerations for implementing this strategy include addressing key issues such supply, standards of education, standards of practice and accreditation. These are highlighted in an article by Salvatori [58] who reported that the actual number of OT personnel delivering OT services in Canada remains unknown and that there are no national standards of education nor accreditation process for OT assistants. CAOT believes that in order to utilize support personnel appropriately, studies on human resource needs for occupational therapy and support personnel are first needed with input from OTs, stakeholders, funders, decision makers and health policy planners [59]. Since there is a lack of competency profiles related to the role, responsibilities, and supervision of assistants, particularly with regards to delivering services in unsupervised community-based settings, the type of workplace setting where this strategy may be implemented should be considered [58].

### Education and Training

Given that 60% of highly rated *Education and Training *strategies targeted rural and remote practices underscores the importance of specific strategies for rural and remote areas in the development of a HHR plan. The need to build on existing mechanisms to expand the availability of rural and remote clinical placements by providing financial and accommodation support was ranked among the top two most important and feasible education strategies for rehabilitation professionals. Not only has this strategy been used as a recruitment tool for rehabilitation students, it has also been reported by Solomon et al. [34] to be effective in retaining OTs and PTs in underserviced Northwestern Ontario communities. Respondents from Solomon et al.'s study reported that the top three benefits of supervising students were that it stimulates thinking, it provides opportunity to contribute to the profession and that it provides access to current information. The reported disadvantages however was that it was time-consuming and students contributed stress to the working environment. Similarly, a two-part study found substantial gaps between financial incentives students deem important in the creation of an appealing clinical placement opportunity and the actual provisions offered to them by Southeastern Ontario communities [60,61]. Although OT and PT students reported that they were more willing to complete a clinical placement in an underserviced community if provided travel stipends, rent-free housing and interprofessional education opportunities, the majority of these incentives were only available to medical students. In addition to training students for rural and remote practice, a longitudinal study reported that perceived opportunity for career development was the most significant factor related to job turnover and regional attrition among physiotherapists working in Northern Ontario [5]. Therefore developing workforce strategies for rehabilitation therapists working in these areas should be among one of the priority areas in HHR planning.

### Professional Development

Our findings indicate that the theme with the largest number of strategies that were considered important and feasible to implement as part of HHR planning was professional development. Many were specific to rural and remote areas. Although the importance of continuous professional development (CPD) in recruitment and retention is well recognized, a Canadian study reported several barriers to its implementation [62]. In the case of OTs employed in public settings in Nova Scotia, Townsend et al. (2006) found that the most powerful deterrent for CPD was the lack of support from workplace policies. Based on their study results, the use of CPD as a recruitment and retention strategy was highly influenced by gender issues, work-life balance, career advancement, working conditions, geographical location, professional versus employer responsibility, and employee benefits. Although occupational therapy is a female-dominated profession, workplace policies did not address issues of gender. For example, therapists in this study indicated that CPD competes with family commitments, therefore these activities are "done largely during personal time, mainly at their own cost, and on top of childcare, eldercare, homemaking and other family responsibilities" [62]. In addition, heavy workloads, lack of salary and career incentives, and lack of policy and funding support are all barriers to CPD. These issues become more pronounced in rural and remote settings because smaller communities often only have one therapist; hence the systemic pressure of workload demands makes it difficult for the therapist to leave patient care. OTs from this study also questioned who was responsible for CPD. Some felt that it was the professional's responsibility while others felt that it was the responsibility of the employer to provide CPD opportunities. The primary limitation employers faced was the lack of financial resources, however giving employees time off without pay was an alternative strategy utilized instead of funding professional development activities. Although there are professional and provincial variations in funding for CPD across Canada, these results are informative in that it highlights the need for employers to consider how workplace policies can affect recruitment and retention strategies.

Limitations of this study should be noted. First, the majority of the strategies were obtained from the grey literature that is not subject to the same scrutiny as the peer-reviewed literature. Second, almost none of the strategies were specifically developed for rehabilitation professionals and in many cases had to be re-worded to fit the rehabilitation context. There is a lack of research on rehabilitation clinicians' perspectives on recruitment and retention strategies; therefore future research should focus on investigating this area. Third, during the face-to-face meeting, bias could have resulted from panelists whose opinion may have influenced others significantly, especially if members came from similar workplace settings. The facilitator of the expert panels however, followed a strict process for managing the discussion and ensured that all panelists were given the opportunity to express their opinions.

Finally, although some strategies such as competitive wage packages, training/growth opportunities and professional development are viewed as both a recruitment and retention incentive, other strategies do not overlap and are appropriate for only one of the two tasks. For example, increasing public awareness of rehabilitation careers, providing rural and remote orientation packages and family relocation programs are only appropriate for attracting a worker while ensuring open and timely communication may be seen as a strategy only for retention. Future research should therefore consider studying recruitment and retention strategies separately so that a distinction between the two can be made.

## Conclusion

This study identified 34 strategies that should be considered as important and feasible for implementation as part of HHR planning for rehabilitation professionals. Although the highest ranked strategies focused on areas of *Quality of Worklife and Work Environment, Financial Incentives and Marketing *and *Professional Development*, key factors that need to be considered in the context of implementation include: workplace setting, geographical location and gender issues. While this is the first study to our knowledge that provides a comprehensive list of recruitment and retention strategies relevant to rehabilitation professionals, more information is needed for the development of a HHR plan. Information on trends in labour force participation as well as knowledge regarding the use and effectiveness of recruitment and retention strategies for rehabilitation professionals is needed. More importantly, the success of implementing and sustaining such strategies requires future research to validate these strategies from the perspective of rehabilitation clinicians and human resource decisions makers (i.e. local government, stakeholders, etc.) so that specific barriers and challenges can be identified.

## Competing interests

The authors declare that they have no competing interests.

## Authors' contributions

DT participated in the design of the study, conducted the literature review, analyzed and interpreted the results and drafted the manuscript.

LMH, AD, DB and KB were involved in providing feedback and editing the content of the manuscript.

MDL recommended participants for the expert panel and was involved in providing feedback and editing the content of the manuscript.

SJ participated in the design of the study, recommended participants for the expert panel, organized and consolidated strategies, interpreted the data and edited the content of the manuscript.

## Pre-publication history

The pre-publication history for this paper can be accessed here:


